# NMR monitoring of the SELEX process to confirm enrichment of structured RNA

**DOI:** 10.1038/s41598-017-00273-x

**Published:** 2017-03-21

**Authors:** Ryo Amano, Kazuteru Aoki, Shin Miyakawa, Yoshikazu Nakamura, Tomoko Kozu, Gota Kawai, Taiichi Sakamoto

**Affiliations:** 1 Department of Life and Environmental Sciences, Faculty of Engineering, Chiba Institute of Technology, 2-17-1 Tsudanuma, Narashino, Chiba 275-0016 Japan; 2 Ribomic Inc., 3-16-13 Shirokanedai, Minato-ku, Tokyo 108-0071 Japan; 3 Department of Basic Medical Sciences, Institute of Medical Science, University of Tokyo, 4-6-1 Shirokanedai, Minato-ku, Tokyo 108-8639 Japan; 4 Research Institute for Clinical Oncology, Saitama Cancer Center, 818 Komuro, Ina, Kitaadachi-gun, Saitama 362-0806 Japan

## Abstract

RNA aptamers are RNA molecules that bind to a target molecule with high affinity and specificity using uniquely-folded tertiary structures. RNA aptamers are selected from an RNA pool typically comprising up to 10^15^ different sequences generated by iterative steps of selection and amplification known as Systematic Evolution of Ligands by EXponential enrichment (SELEX). Over several rounds of SELEX, the diversity of the RNA pool decreases and the aptamers are enriched. Hence, monitoring of the enrichment of these RNA pools is critical for the successful selection of aptamers, and several methods for monitoring them have been developed. In this study, we measured one-dimensional imino proton NMR spectra of RNA pools during SELEX. The spectrum of the initial RNA pool indicates that the RNAs adopt tertiary structures. The structural diversity of the RNA pools was shown to depend highly on the design of the primer-binding sequence. Furthermore, we demonstrate that enrichment of RNA aptamers can be monitored using NMR. The RNA pools can be recovered from the NMR tube after measurement of NMR spectra. We also can monitor target binding in the NMR tubes. Thus, we propose using NMR to monitor the enrichment of structured aptamers during the SELEX process.

## Introduction

Aptamers are nucleic acid molecules that are selected from large random sequence libraries (10^14^–10^15^ unique/different sequences) based on their high affinity for target molecules by a process known as Systematic Evolution of Ligands by EXponential enrichment (SELEX)^1–9^. Similar to antibodies, aptamers recognize a broad range of target molecules (e.g., nucleotides, cofactors, amino acids, peptides, polysaccharides, proteins, whole cells, viruses, and single-celled organisms) with high affinity and specificity and hence are expected to serve as therapeutic agents^10–12^. Although antibodies are the most commonly used molecular tool for recognizing target molecules, the use of aptamers has some advantages over antibodies, including greater thermal stability, lower immunogenicity, and greater ease of production^13, 14^.

SELEX for obtaining RNA aptamers comprises selection steps (target binding, separation of target-bound RNAs from unbound RNAs) and amplification steps (reverse transcription, PCR amplification and transcription, and purification of selected RNAs). To obtain high affinity aptamers from random sequence libraries, the SELEX process should be repeated several times. Given that the selection conditions are critical to obtaining high-affinity aptamers and are dependent on the characteristics of target molecules, a great deal of time is spent on trial and error to optimize the conditions. Thus, monitoring the progression of SELEX is important for allowing early intervention and adjustment of the selection conditions.

Various approaches for monitoring the SELEX process have been reported^15–33^. The most straightforward monitoring method involves the assessment of sequence enrichment in RNA pools, a technique made possible by the advent of high throughput sequencing (HTS) technology^15–20^. However, HTS is expensive, and sample preparation for HTS is time-consuming. Another effective way of monitoring is to assess the average affinity of the RNA pools to the target molecule^16, 23–33^. Several such assessment methods are available, including surface plasmon resonance (SPR), the electrophoretic mobility shift assay (EMSA), and the filter-binding assay. Furthermore, fluorescence-activated cell sorting (FACS) has recently been applied for monitoring the evolution of nucleic acid pools^32, 33^. However, SPR requires immobilization of the target molecules or RNA pools onto a sensor chip. To conduct EMSA, the filter-binding assay and FACS, the RNA pools or target molecules must be labeled with tags such as fluorophores or radioisotopes. Such immobilization and labeling are time consuming and sometimes change the structure and binding properties of the RNA or target molecules. Therefore, an efficient, simple, and rapid approach for monitoring RNA pool enrichment during SELEX is needed.

**Figure 1:**
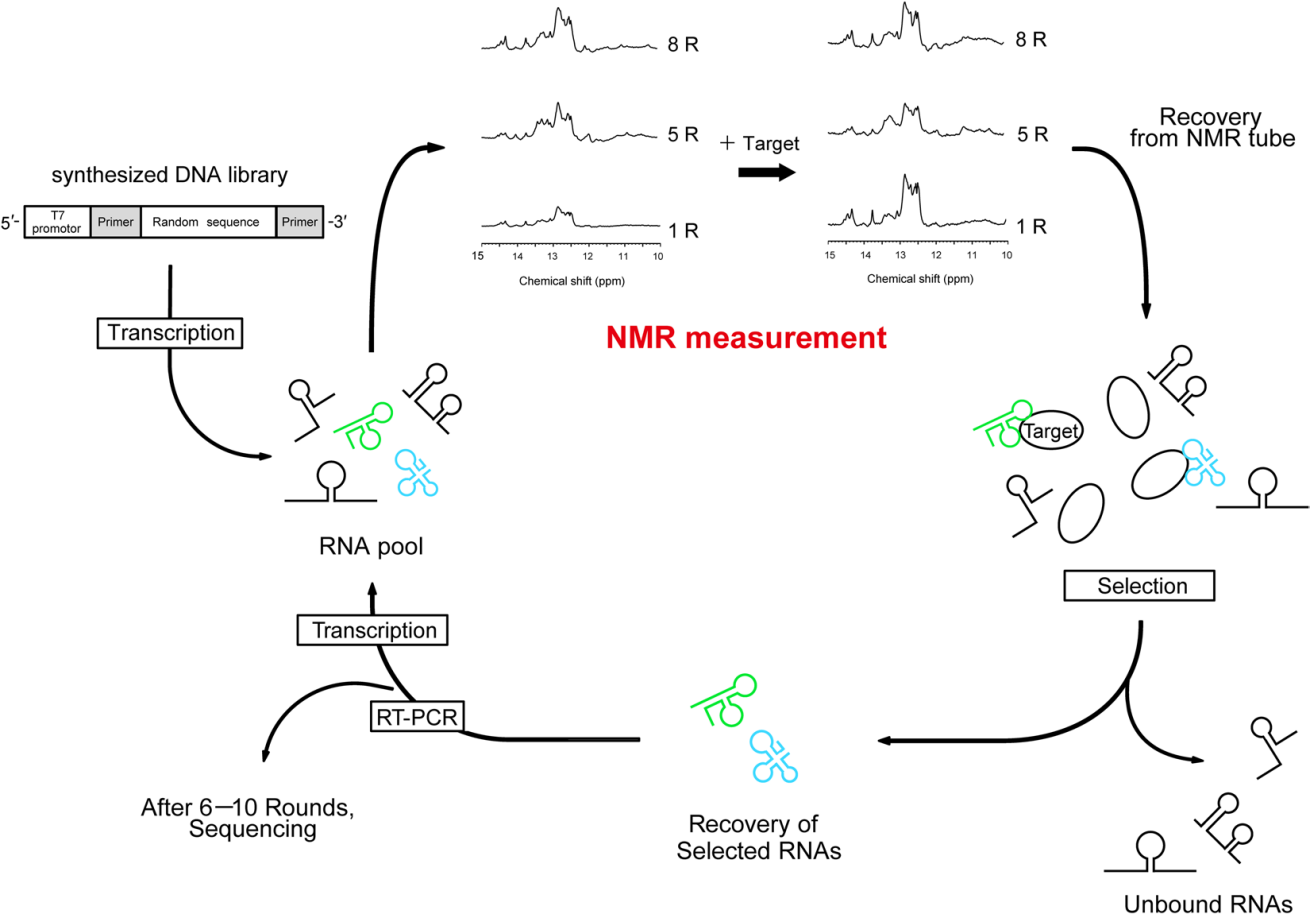
Schematic outline of NMR monitoring of the SELEX process. During each round of SELEX, imino proton signals of RNA pools and RNA binding to target proteins are monitored by NMR and then subjected to selection. Only 1 or 2 hours are required for measurement of the NMR spectra. The addition of target molecules can be skipped.

Nuclear magnetic resonance (NMR) spectroscopy is an excellent tool for analyzing the structures of RNA molecules^34–41^. Imino proton signals of the guanosine and uridine residues observed between 10 and 15 ppm contain valuable information about base-pairing in the RNA molecule, as these signals are observable when the imino protons are involved in hydrogen bonding or protected from exchange with the bulk solvent water^42–44^. Thus, Watson–Crick base pairs (G-C and A-U), non-Watson–Crick base pairs (e.g., G-U or G-A), and G-quartets are detectable by imino proton spectrum analysis of RNA. Given that RNA aptamers are known to adopt characteristic conformations and recognize target molecules, imino proton spectra of RNA pools also can provide useful information about the enrichment and structures of aptamers. Furthermore, one-dimensional (1D) imino proton spectra of RNA pools can be measured in just 1 hour, without immobilization or labeling. As shown in Fig. 1, imino proton spectra of RNA pools can be measured after transcription and purification without any additional preparation for NMR measurement; RNA pools then can be recovered from the NMR tubes and directly used for selection.

In a previous study, we obtained high-affinity RNA aptamers against the AML1 Runt domain (RD) after 8 rounds of SELEX^45^. To evaluate NMR monitoring for the enrichment of RNA aptamers during the SELEX process, we measured 1D imino proton spectra of the RNA pools obtained in our previous study. Furthermore, we measured the NMR spectra of initial RNA pools that were obtained using the other primer-binding sequence sets to compare with that of the previous study. Here we propose the use of NMR to monitor changes in the RNA pool composition and the enrichment of structured aptamers during SELEX.

## Results

### Comparison of 1D imino proton spectra of RNA pools during SELEX

**Figure 2:**
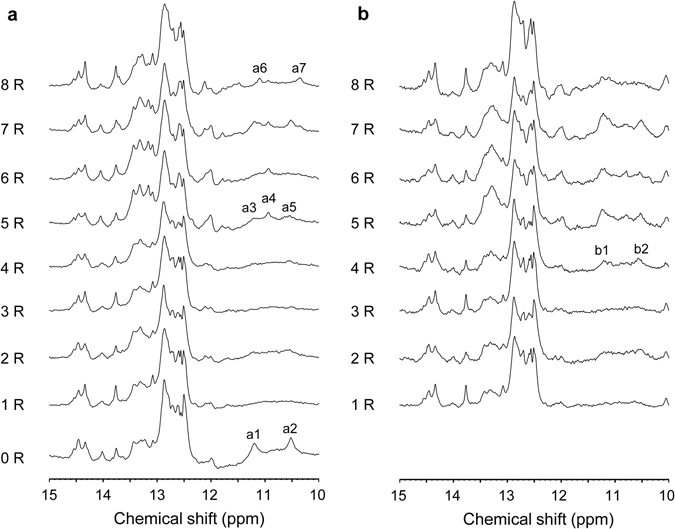
1D imino proton spectra of RNA pools in the absence and presence of RD. 1D imino proton spectra of 0–8R RNA pools of SELEX in the absence (**a**) and presence (**b**) of RD.

To evaluate NMR monitoring during SELEX, we measured 1D imino proton spectra of the initial RNA pool (0R) and RNA pools from the 1^st^ to 8^th^ round (1R–8R) of SELEX against RD (Fig. 2a). Numerous signals were observed in the spectrum of the 0R RNA pool, indicating that this pool included a large number of structured RNAs. Although no large change in the NMR spectra was observed over 8 rounds of SELEX, some signals did change; such a change was clearly seen at 10–12 ppm (Supplementary Fig. S1a). Signals a1 and a2 at 10–12 ppm disappeared after the first round of SELEX, and new signals a3, a4, and a5 appeared at 10–12 ppm after 5R of SELEX. Furthermore, intensities of the signals a3, a4, and a5 were decreased and those of new signals a6 and a7 were increased over rounds 5R to 8R. These signals at 10–12 ppm indicate the presence of RNAs forming non-Watson–Crick base pairs in the RNA pools.

As a control experiment, we performed Neutral SELEX, which skips the selection steps, and measured the imino proton spectra of the RNA pools (Supplementary Fig. S2). Comparison of these imino proton spectra shows little change through 8 rounds of Neutral SELEX, indicating that changes in the imino proton spectra were caused by the SELEX selection steps.

Next, we added RD to the RNA pools and concentrated the samples using ultrafiltration membranes. However, the NMR sample for the 0R RNA pool with RD could not be prepared due to aggregation of free RD. Thus, we measured the 1D imino proton spectra of RNA pools 1–8R in the presence of RD (Fig. 2b) and compared the spectra between without and with RD (Supplementary Fig. S3). Comparison of the spectra for RNA pools 1–3R shows that the imino proton signals did not change upon addition of RD. However, in the case of the 4R RNA pool, new signals b1 and b2 were observed at 10.5–12 ppm upon addition of RD (Supplementary Fig. S1b). Furthermore, in the spectra of the 5–8R RNA pools, these signals changed dramatically upon addition of RD. These results suggest that minor aptamer enrichment begins at 4R, with marked enrichment at 5–8R.

### SPR analysis of RD binding of RNA pools

**Figure 3:**
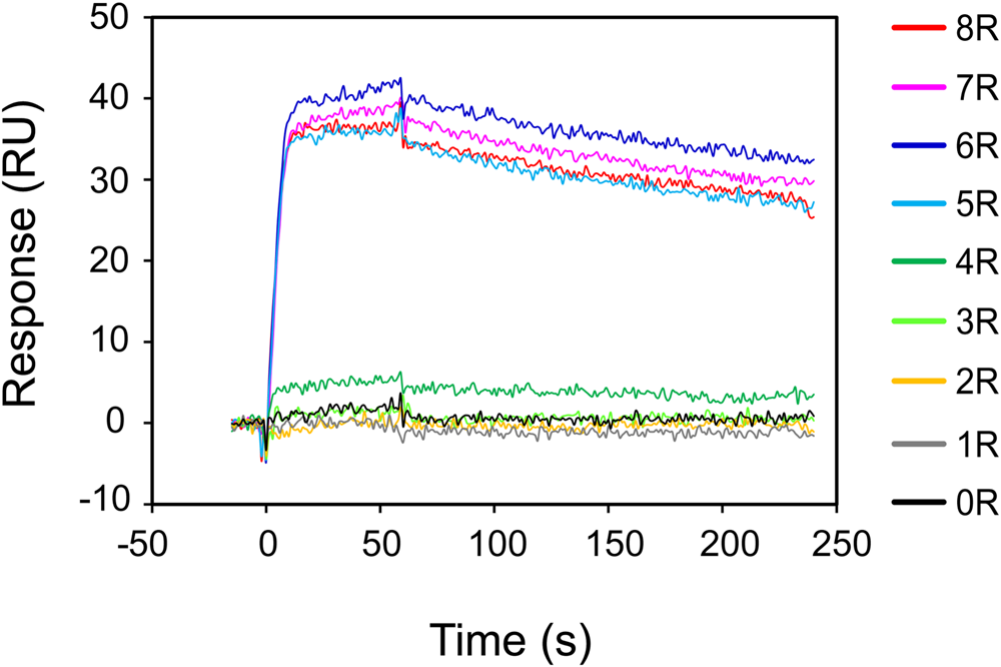
SPR analysis of the affinity of RNA pools from SELEX. SPR sensorgrams of interactions between RD and 0–8R RNA pools during SELEX. Sensorgrams of 0–8R pools are shown in black, gray, yellow, light green, green, cyan, blue, magenta, and red, respectively. Final concentration of RD was 10 nM.

We examined the affinity of the RNA pools using SPR. The 0–3R RNA pools showed no affinity for RD, while the 4R pools showed slight affinity and the 5–8R pools showed significant affinity (Fig. 3). This result is consistent with the NMR data showing that imino proton signals at 10–12 ppm in the presence of RD appeared at 4R and changed markedly at 5–8R (Fig. 2b).

### HTS analysis of RNA pools during SELEX

**Figure 4:**
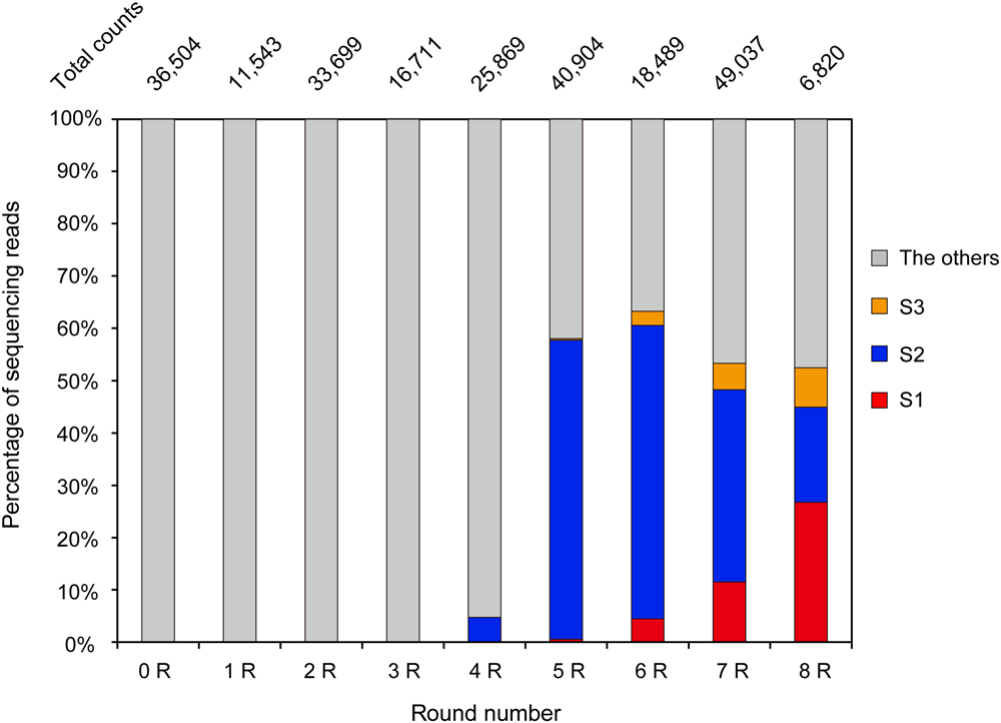
The fraction of S1, S2, and S3 in the sequencing reads from 0–8R RNA pools from SELEX. Total counts are the numbers of total reads obtained by HTS. S1, S2, S3, and others are shown in red, blue, orange, and gray bars, respectively.

HTS analysis was performed on the 0–8R RNA pools. Sequencing reads were sorted into sets by barcode sequence and processed based on the primer-binding sequences, resulting in the final sequencing reads for each round (0R; 36,504 clones; 1R, 11,543 clones; 2R, 33,699 clones; 3R, 16,711 clones; 4R, 25,869 clones; 5R, 40,904 clones; 6R, 18,489 clones; 7R, 49,037 clones; and 8R, 6,820 clones) (Fig. 4). Sequences were aligned and ranked by fraction of the total (% of RNA sequence reads).

HTS data were compared with the sequences of the 36 clones in the 8R RNA pool that were determined using the capillary sequencing method^45^. The fractions of each aptamer were determined as follows: S1, 27.8%; S2, 22.2%; S3, 16.7%. The predicted secondary structure and dissociation constant of the aptamers are shown in Supplementary Fig. S4. The HTS data for this pool revealed similar aptamer fractions (S1, 26.7%; S2, 18.2%; S3, 7.2%). In the HTS data of early rounds, aptamers S1, S2, and S3 were not detected in the 0–2R RNA pools, and only one sequence of S2 was read out from the 3R RNA pool, indicating that the total number of sequences in pools 0–3R was greater than that of the total sequencing reads. S2 was slightly enriched (4.7%) at 4R. At 5R, S2 was markedly enriched (57.2%), whereas the frequencies of S1 and S3 were extremely low (less than 1%). Through 6R and 7R, the fraction of S2 decreased, whereas that of S1 and S3 increased. Finally, the fraction of S1 increased over that of S2 at 8R. This HTS result is consistent with the NMR data showing that imino proton signals at 10–12 ppm in the absence of RD appeared at 5R and gradually changed from 6R through 8R.

### Comparison of imino proton spectra between RNA pools and the isolated aptamers

**Figure 5:**
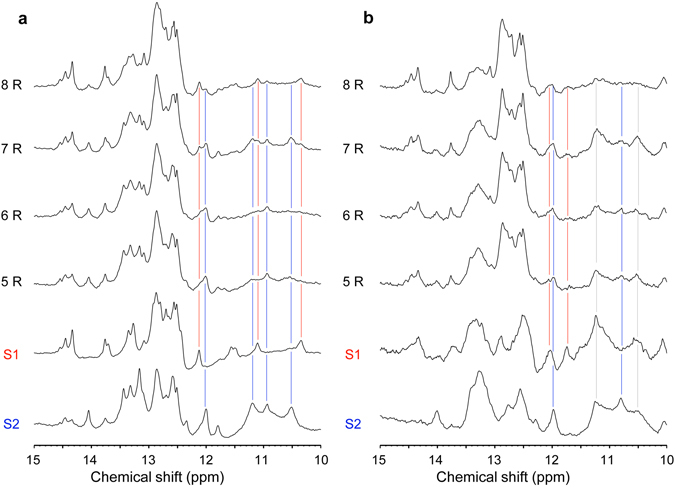
Comparison of 1D imino proton spectra between RNA pools from SELEX, S1, and S2. Comparison of 1D imino proton spectra of 5–8R RNA pools with those of S1 (red) and S2 (blue) in the absence (**a**) and presence (**b**) of RD. Red and blue lines indicate discriminative imino proton signals of S1 and S2, respectively; gray lines indicate those common to both S1 and S2.

We focused on the aptamers S1 and S2, which were sufficiently abundant to observe them in the pool, although other aptamers that showed higher affinity to RD than these two aptamers were obtained in a previous study^45^. Given that HTS data revealed that the fraction of S1 and S2 was high at 5–8R, we prepared isolated S1 and S2 aptamers and compared the imino proton spectra of 5–8R RNA pools with those of S1 and S2 (Fig. 5). The spectra of the isolated S1 and S2 changed with signals broadening upon RD binding (Supplementary Fig. S5). In the absence of RD (Fig. 5a), the signals at approximately 10.3, 11.1, and 12.1 ppm observed in the spectrum of S1 were similar to those observed for the 8R RNA pool. These signals could not be clearly identified at 5–7R because of the low content of S1. On the other hand, the signals at approximately 10.5, 10.9, 11.2, and 12.0 ppm observed for S2 were also observed in the spectra of 5–7R. In the presence of RD (Fig. 5b), the signals at approximately 11.7 and 12.1 ppm observed in the spectrum of S1 were also observed for the 8R RNA pool. The signals at approximately 10.8 and 12.0 ppm observed for S2 were also observed in the spectra of 5–7R. Furthermore, the signals at approximately 10.5 and 12.0 ppm observed for both S1 and S2 were also observed in the spectra of 5–7R. Thus, the NMR spectra of the RNA pools reflect the fraction of RNA aptamers in the RNA pools.

### Effect of primer-binding sequence on NMR spectra of RNA pools

To determine whether the NMR spectrum of the 0R RNA pool was derived from the primer-binding sequence, we predicted the secondary structure of the 0R RNA pool using vs_subopt^46, 47^. Although a short stem containing a bulged A residue was predicted, a stable stem structure cannot form at the primer-binding sequences (Supplementary Fig. S6a). Next, we prepared RNA containing only primer-binding sequences, with no 40-nucleotide random sequences (Supplementary Fig. S6b), and measured the 1D imino proton spectrum (Supplementary Fig. S6d). The imino proton spectra were quite similar between the 0R RNA pool and the primer-binding sequence RNA, although some signals of the primer-binding sequence RNA were weaker than those of the 0R RNA pool. Differences between the two spectra might arise from structural differences of the primer-binding region between the 0R RNA and the primer-binding sequence RNA, which is constructed by directly conjugating primer-binding sequences. However, the similarity of the two spectra indicated that the NMR spectrum of the 0R RNA pool was derived from the primer-binding sequence. We prepared a pool of poly (A-U) RNAs containing a 40-nucleotide random sequence flanked by an A- or U-rich sequence for primer binding (Supplementary Fig. S6c) and measured the imino proton spectrum of the RNA pool (Supplementary Fig. S6d). A large signal that is typical of A-U base pairs was observed at 13.5 ppm. Other signals were barely visible. Furthermore, to confirm the primer-binding sequence dependence of the spectra of 0R RNA pools, we measured the imino proton spectra of the other 0R pools (RNA pools_2, 3, and 4), which contain different primer-binding sequences (Supplementary Fig. S7). These spectra were significantly different among them. Therefore, comparison of these spectra shows that the spectrum of the random RNA pool is significantly affected by the primer-binding sequences.

## Discussion

Imino proton signals of Watson–Crick base pairs and non-Watson–Crick base pairs typically are observed at 12–15 ppm and 10–12 ppm, respectively. Thus, the NMR results indicate that aptamers S1 and S2 form both Watson–Crick base pairs and non-Watson–Crick base pairs. According to the computational prediction of secondary structure, the aptamers contain stem and loop structures (Supplementary Fig. S4), suggesting that the loop regions of S1 and S2 should adopt characteristic conformations containing non-Watson–Crick base pairs. These characteristic conformations might be important for the specific binding of the aptamers to RD.

The 0R RNA pool spectrum showed multiple imino proton signals, although secondary structure prediction showed that the primer-binding sequences do not adopt stable structure by themselves (Supplementary Fig. S6). The imino proton spectra were quite similar between the 0R RNA pool and primer-binding sequence RNA, indicating that the NMR spectrum of the 0R RNA pool was derived from the primer-binding sequence. In contrast, no signals were observed from 40-nucleotide random sequences, as the primer-binding sequences were designed to adopt stem structures in the poly (A-U) RNA pool, suggesting that the structural diversity in the poly (A-U) RNA pool would hardly be affected by the primer-binding sequences. However, the use of a poly (A-U) RNA pool is not practicable to SELEX because of the low efficiency of the amplification step. Furthermore, the spectra of the RNA pools_2, 3, and 4 containing the other primer-binding sequence sets were significantly different among them (Supplementary Fig. S7). These results indicate that the design of the primer-binding sequence affects structural diversity in the initial RNA pool, which would be important for success in SELEX experiments. Further investigation of the influence of primer-binding sequence on the structural diversity of the RNA pool using NMR would enhance the optimization of SELEX efficiency. In previous studies, randomized oligonucleotide libraries with no primer-binding sequences were used for SELEX (“Tailored-SELEX”)^48, 49^. Tailored-SELEX can remove the bias of the primer-binding sequence, although it is time-consuming because the primer-binding sequences are ligated to the pool after the selection step.

The spectrum of the initial RNA pool (0R) without RD reveals imino proton signals at 10–12 ppm, which disappeared in the spectrum of the 1R RNA pool (Fig. 2a). This change in the NMR spectrum indicates that the population of RNA molecules that form non-Watson–Crick base pairs decreased, although the reason for the dwindling of such RNA molecules is unknown. New imino proton signals appeared in the 10–12 ppm region in the spectrum of the 5R RNA pool; these signals changed over the course of 6R through 8R (Fig. 2a). Similarly, the imino proton signals in the 10.5–12 ppm region appeared at 4R in the RNA pool with RD; these signals changed over the course of 5R through 8R (Fig. 2b). In parallel, the binding affinity and the RNA pool composition also changed dramatically at 5R (Figs 3 and 4). Furthermore, comparison of the signals at 10–12 ppm in the spectra of the 5–8R RNA pools with those of S1 and S2, which were highly enriched in 5–8R, reveals that the NMR spectra of RNA pools reflects the degree of RNA aptamer enrichment. As noted above, the NMR data are consistent with the SPR and HTS data. Therefore, these data suggest that NMR is suitable for monitoring the enrichment of aptamers in RNA pools during the SELEX process.

The fraction of S2 gradually decreased from 5R to 8R, whereas that of S1 incrementally increased and exceeded that of S2 at 8R, even though S2 showed higher binding affinity than S1 (Supplementary Fig. S4). Although this RNA composition change could not be explained by the NMR and HTS data, one possible explanation is bias in the efficiency of transcription or reverse-transcription PCR in SELEX^50^. The sequence of S1 might be more suitable than S2 for amplification in the SELEX process.

SPR is more useful for our goals than NMR, because we perform SELEX to obtain high-affinity aptamers. However, even with the use of NMR, binding of RNA pools to target molecules could be monitored by simply adding target molecules to the NMR tube (Fig. 2b, Supplementary Fig. S1b and S3). In this study, we could not add concentrated RD directly but instead added it using ultrafiltration membranes. Changes in the imino proton spectra of free RNA pools, in the absence of target molecules, provides information about the enrichment of the structured RNA aptamers (Fig. 2a and Supplementary Fig. S1a). Thus, the addition of target molecules can be avoided if they are valuable. HTS is more informative than NMR with respect to sequence enrichment monitoring, as HTS provides detailed sequence information. However, NMR provides structural information about the RNA pool. Many aptamers adopt a G-quadruplex conformation^51^. NMR monitoring would be suitable for detecting G-quadruplexes because unique imino proton signals can be observed at 10–12 ppm^52–54^. Finally, the most important point we would like to emphasize is that NMR monitoring is simple and fast. While SPR and HTS require 2–3 days for sample preparation and 1 day for measurement, a 1D imino proton spectrum can be measured within 1 or 2 hours without extra sample preparation. After the RNA pools are transcribed and purified, they can be injected into NMR tubes. After NMR measurement, RNA pools can be recovered from the NMR tubes and directly used for selection. A recently-proposed method known as INTT^34^ involves the transcription of RNAs in an NMR tube, followed by NMR measurement without RNA purification. Combining INTT with SELEX might enhance the rapid screening of aptamers. As described here, NMR is useful for monitoring the enrichment of aptamers in RNA pools during SELEX. Although the NMR spectra would be different for different SELEX experiments, the new imino proton signals of RNA aptamers will appear whenever the aptamers are enriched. Furthermore, the change in the imino proton spectra upon target protein binding would be more clearly differentiated between the aptamer-enriched and -non-enriched pools. Thus, we would confirm aptamers enrichment by monitoring the change in the NMR spectra for a new SELEX experiment. Furthermore, NMR monitoring could be useful for DNA aptamers, enabling imino proton signals to be observed for structured DNAs. We believe that NMR monitoring accelerates the discovery and identification of high-quality aptamers.

## Methods

### Neutral SELEX

As a control experiment, SELEX was performed as described previously^45^ except that the selection step was omitted, a procedure referred to as “Neutral SELEX”^15^.

### Sample preparation

RNA pools and aptamers (S1 and S2) were prepared as described previously^45^. The poly (A-U) RNA pool was prepared using a DNA template (5′-CGGAAAAAAAAAAAAAAAAAAAAG-40N-ATTTTTTTTTTTTTTTTTTTTCCCTATAGTGAGTCGTATTA-3′; the T7 promoter sequence is underlined, and 40N represents 40 nucleotides (nt) of random sequence and primers (Fwd1; 5′-TAATACGACTCACTATAGGGAAAAAAAAAAAAAAAAAAAAT-3′ and Rev1; 5′-CGGAAAAAAAAAAAAAAAAAAAAG-3′) (Hokkaido System Science Co., Ltd, Sapporo, Japan). The RNA pools 2, 3, and 4 were prepared using a DNA template (5′-CAAGGAGCGACCAGAGG-N40-TGGCATCCTTCAGCCCTATAGTGAGTCGTATTA-3′, 5′-GGGTGTTAGCTGTTAGTATC-N40-GGTACGATCAGCTAGCCCTATAGTGAGTCGTATTA-3′, and 5′-AGATGGCACGACTCGG-N40-TGGCATCCTTCAGCCCTATAGTGAGTCGTATTA-3′, respectively) and primers (Fwd2, 3, and 4; 5′-TAATACGACTCACTATAGGGCTGAAGGATGCCA-3′, 5′-TAATACGACTCACTATAGGGCTAGCTGATCGTACC-3′ and 5′-TAATACGACTCACTATAGGGCTGAAGGATGCCA-3′, and Rev2, 3, and 4: 5′-CAAGGAGCGACCAGAGG-3′, 5′-GGGTGTTAGCTGTTAGTATC-3′, and 5′-AGATGGCACGACTCGG-3′, respectively). All RNA samples, including the RNA pools, were purified by phenol/chloroform extraction, ethanol precipitation, and gel filtration using Micro Bio-Spin columns P-30 (Bio-Rad, Hercules, CA, USA). AML1-RD was prepared as described previously^45^. All RNA samples were annealed by heating at 95 °C for 5 min followed by snap-cooling on ice and dissolving in NMR buffer (20 mM sodium phosphate, pH 6.5) containing 300 mM potassium chloride, 2 mM magnesium chloride, and 5% D_2_O. The final concentration of all RNA samples was 0.1 mM. Following NMR measurements, RNA samples recovered from the NMR tubes (Shigemi, Tokyo, Japan) were mixed with purified RD. Subsequently, the mixtures were dissolved in NMR buffer containing 1 mM deuterated DTT and then concentrated to 0.05 mM using filtration membranes with a molecular weight cut-off of 3000–5000 (e.g., Vivaspin 2 from Sartorius AG, Gottingen, Germany).

### NMR spectroscopy

NMR spectra were measured using Bruker AVANCE 600 spectrometers (Bruker Biospin, Billerica, MA, USA). 1D imino proton spectra were recorded using the jump-and-return scheme for water suppression at probe temperatures of 298 K^55^. A total of 1024 scans were made (approximately 45 min). NMR data were processed using the software Topspin 3.5 (Bruker Biospin).

### Surface plasmon resonance (SPR) experiment

SPR experiments were performed as described previously using a BIAcore X instrument (GE Healthcare, Sunnyvale, CA, USA)^45, 56^. RD was dissolved in SPR buffer (20 mM sodium phosphate [pH 6.5], 2 mM magnesium acetate, 0.1% Tween 20, and 1 mM DTT, containing 300 mM potassium acetate) at a concentration of 10 nM.

### High-throughput sequencing (HTS)

HTS was performed using an Ion PGM sequencer (Life Technologies, Carlsbad, CA, USA). The cDNAs from each round of SELEX were amplified by PCR with Ex Taq polymerase (TaKaRa, Shiga, Japan) using specific fusion primers (T7fwd, 5′-CCATCTCATCCCTGCGTGTCTCCGACTCAGC–Barcode–Barcode Adaptor–TAATACGACTCACTATAG-3′; and Rev5, 5′-CCTCTCTATGGGCAGTCGGTGAT–CTCTCATGTCGGCCGTTA-3′) as recommended by Life Technologies, followed by Exonuclease I treatment (New England BioLabs, Medford, MA, USA). The PCR products were purified by phenol/chloroform extraction and then precipitated with ethanol. The 1.3 amol of each products (1.2 μL of 1.1 pM products) was mixed and then amplified by clonal emulsion PCR using an Ion PGM Template OT2 200 kit (Life Technologies) followed by generation and enrichment of template-positive Ion Sphere particles, using an Ion OneTouch 2 and Ion OneTouch ES System (Life Technologies) according to the manufacturer’s instructions. HTS was performed using an Ion PGM Sequencing 200 Kit v2 and an Ion 314 Chip. The resulting HTS data were uploaded to the Ion PGM Torrent Server to process base calling and evaluate the sequence quality. The acceptance criterion was a quality score >Q20 (99% sequence accuracy). Sequence clustering was conducted using the Aptamer Clustering 2.0 software (Life Technologies) as follows. In brief, the nucleotide sequences carrying the intact primer sequences were extracted and clustered by identity of the randomized 40-nucleotide region. Both extremely short and long sequences, such as the primer dimers and unexpectedly-generated longer PCR products, were excluded from the clustering. The cut-off values for minimum and maximum nucleotide lengths of the randomized sequence region for inclusion in clustering were 30 nt and 50 nt, respectively. The isolated sequences were processed using Microsoft Excel.

## Electronic supplementary material

Supplementary Information
