# Supplementary material for: NMR monitoring of the SELEX process to confirm enrichment of structured RNA
Source: Sci Rep. 2017 Mar 21;7:283. doi: 10.1038/s41598-017-00273-x (PMC5428055; doi:10.1038/s41598-017-00273-x)
Supplement: Supplementary file 1 — Supplementary Information [file 41598_2017_273_MOESM1_ESM.pdf]

Supplementary Information

NMR monitoring of the SELEX process to confirm enrichment of structured RNA

Ryo Amano[1], Kazuteru Aoki[2], Shin Miyakawa[2], Yoshikazu Nakamura[2, 3], Tomoko Kozu[4], Gota Kawai[1], and Taiichi Sakamoto[1*]

[1]Department of Life and Environmental Sciences, Faculty of Engineering, Chiba Institute of Technology, Chiba 275-0016, Japan
[2]Ribomic Inc., Tokyo 108-0071, Japan
[3]Department of Basic Medical Sciences, Institute of Medical Science, University of Tokyo, Tokyo 108-8639, Japan
[4]Research Institute for Clinical Oncology, Saitama Cancer Center, Saitama 362-0806, Japan
[*] taiichi.sakamoto@p.chibakoudai.jp

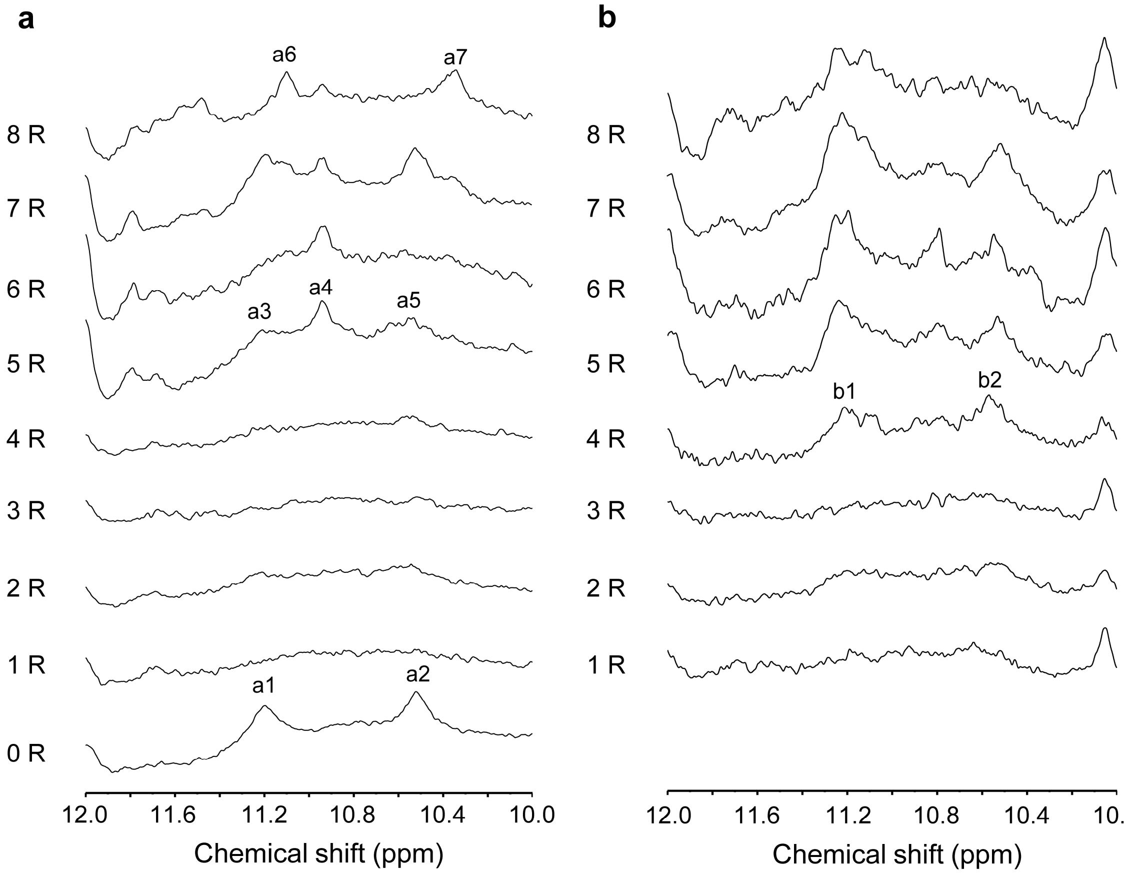

Supplementary Figure S1. 1D imino proton spectra (10–12 ppm) of RNA pools in the absence and presence of RD. 1D imino proton spectra of 0–8R RNA pools from SELEX in the absence (**a**) and presence (**b**) of RD.

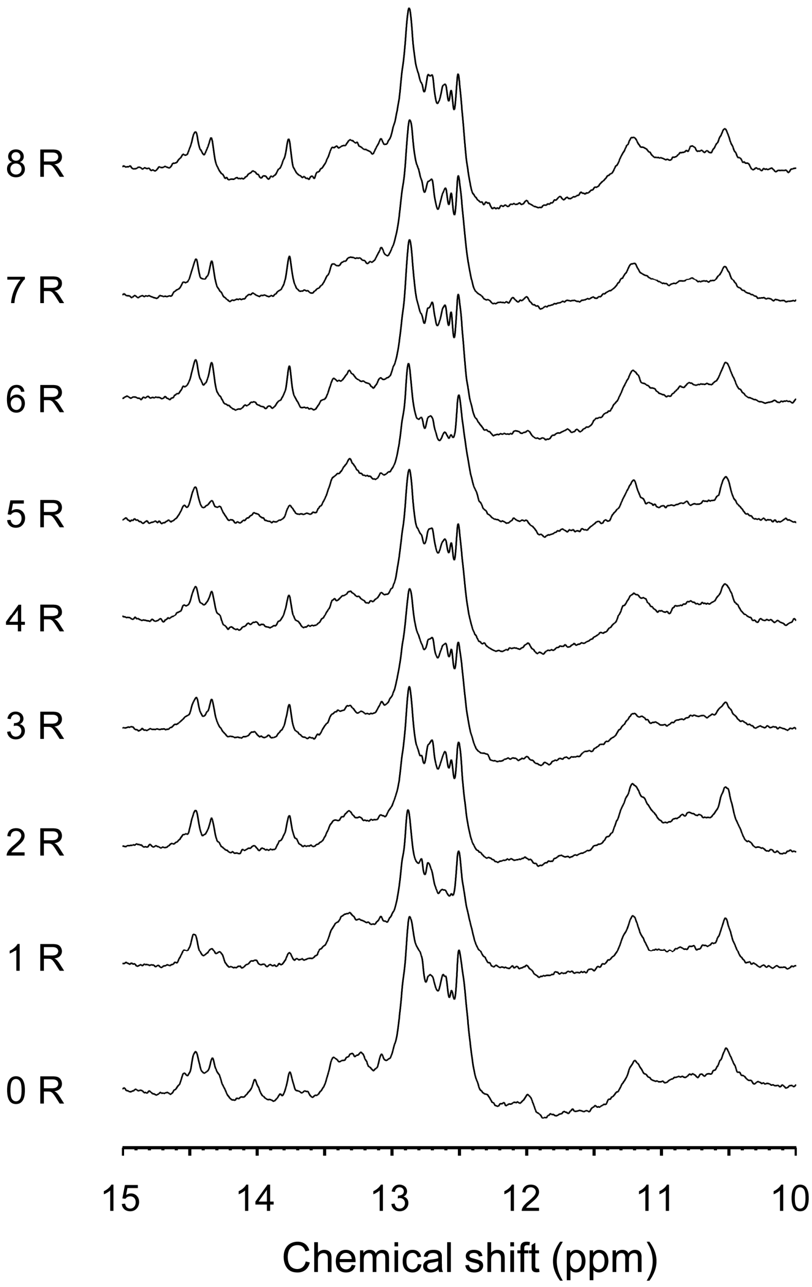

Supplementary Figure S2. 1D imino proton spectra of RNA pools from Neutral SELEX.

1D imino proton spectra of 0–8R RNA pools from Neutral SELEX in the absence of RD.

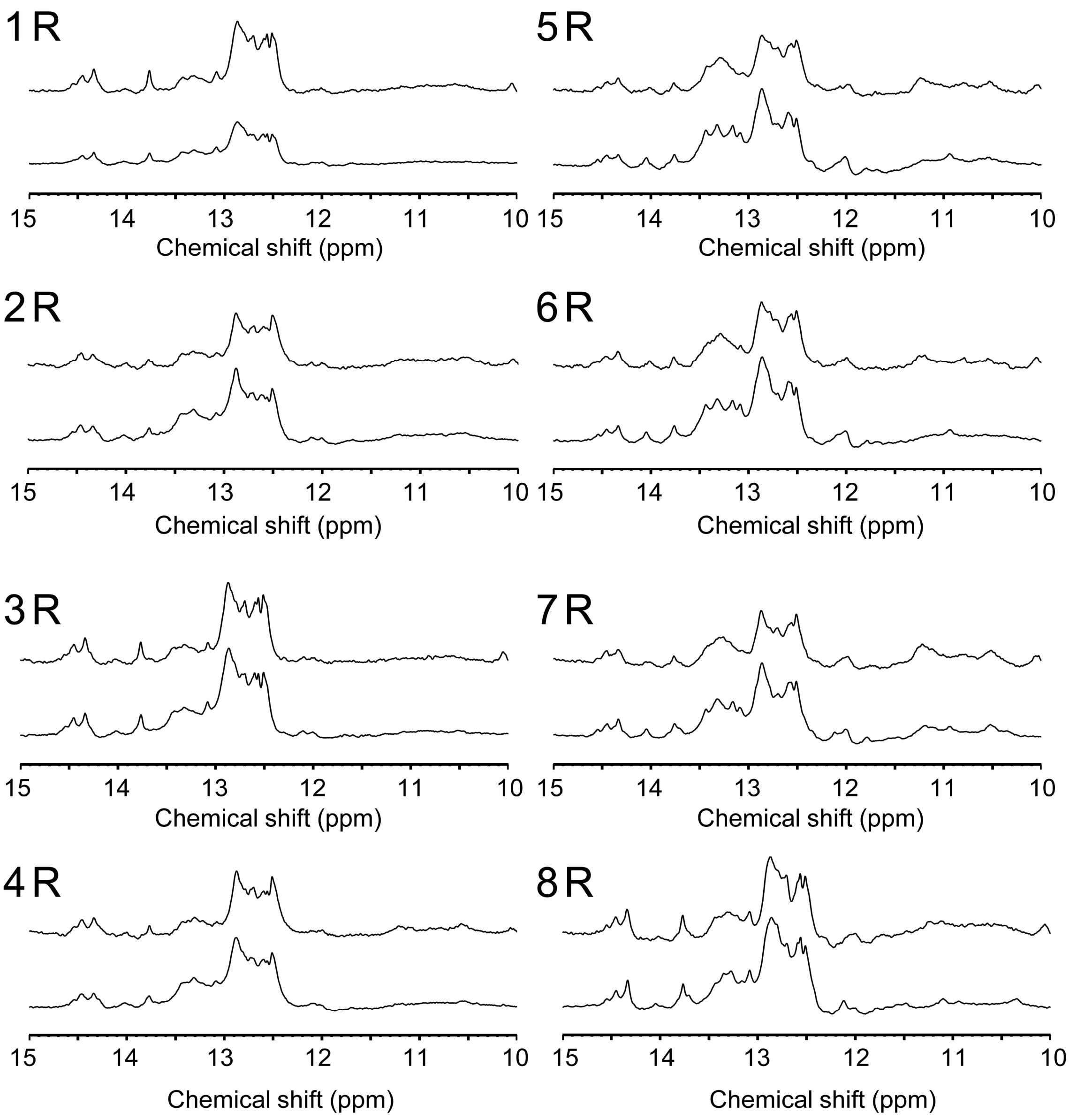

Supplementary Figure S3. Comparison of 1D imino proton spectra of RNA pools from SELEX in the absence and presence of RD. 1D imino proton spectra of 0–8R RNA pools from SELEX in the absence (bottom) and presence (top) of RD.

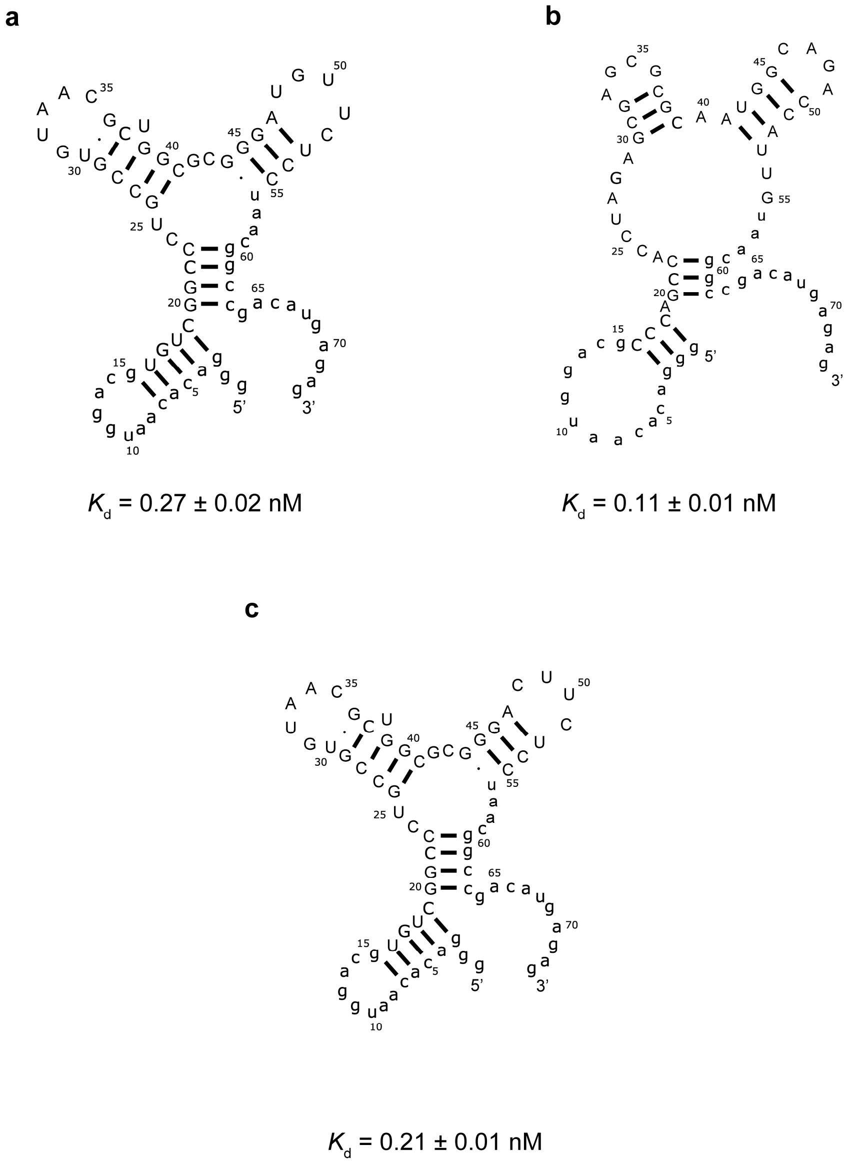

Supplementary Figure S4. The predicted secondary structures and K_d values of S1, S2, and S3.

The secondary structures of S1 (**a**), S2 (**b**), and S3 (**c**) were predicted using vs_subopt.[1, 2] Lower-case letters indicate nucleotides derived from the primer-binding sequences. The K_d values were previously reported.[3]

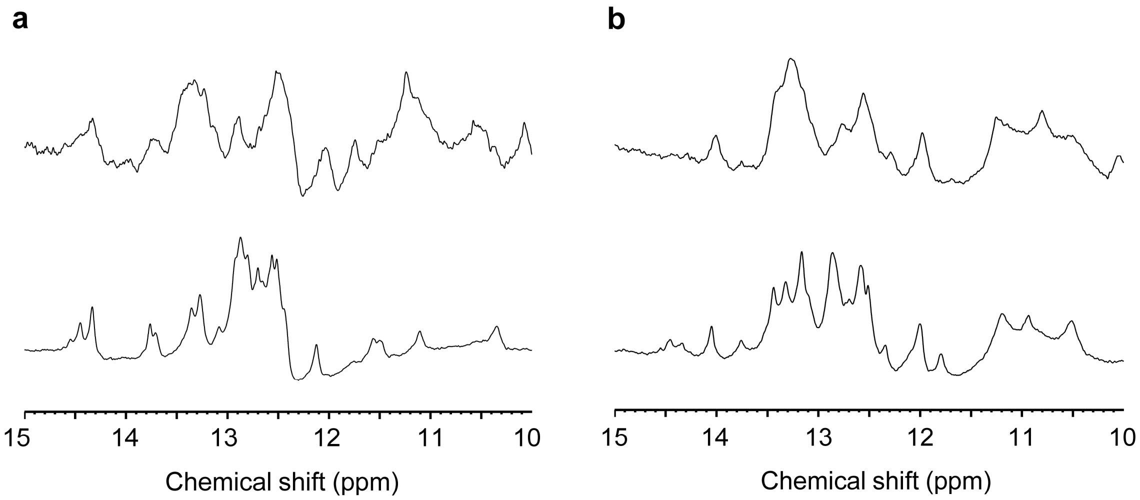

Supplementary Figure S5. Comparison of 1D imino proton spectra of S1 and S2 in the absence and presence of RD. 1D imino proton spectra of S1 (**a**) and S2 (**b**) in the absence (bottom) and presence (top) of RD.

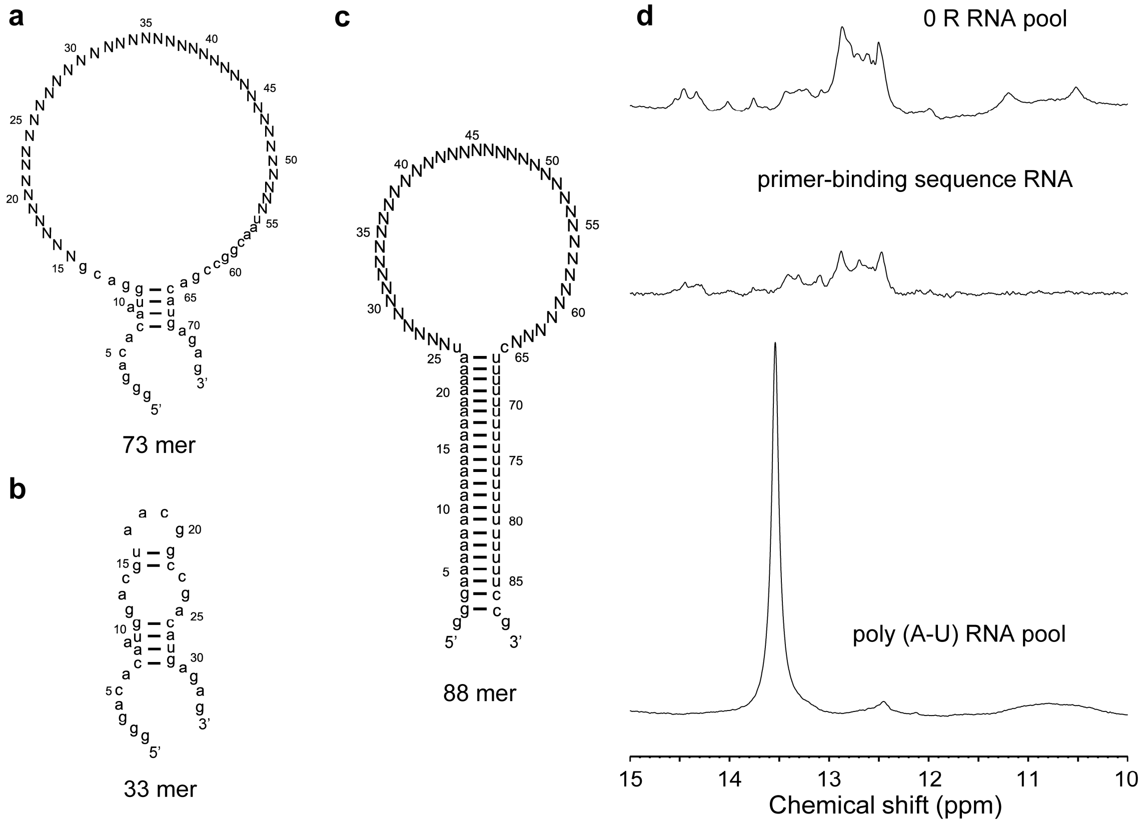

Supplementary Figure S6. Comparison of 1D imino proton spectra between the 0R RNA pool, RNA primer-binding sequences, and poly (A-U) RNA pool. The predicted secondary structure of the 0R RNA pool (**a**), primer-binding sequences RNA (**b**), and poly (A-U) RNA pool (**c**). Lower-case letters indicate nucleotides derived from the primer-binding sequences. 1D imino proton spectra of the 0R RNA pool (top), primer-binding sequence RNA (middle), and (A-U) RNA pool (bottom) (**d**).

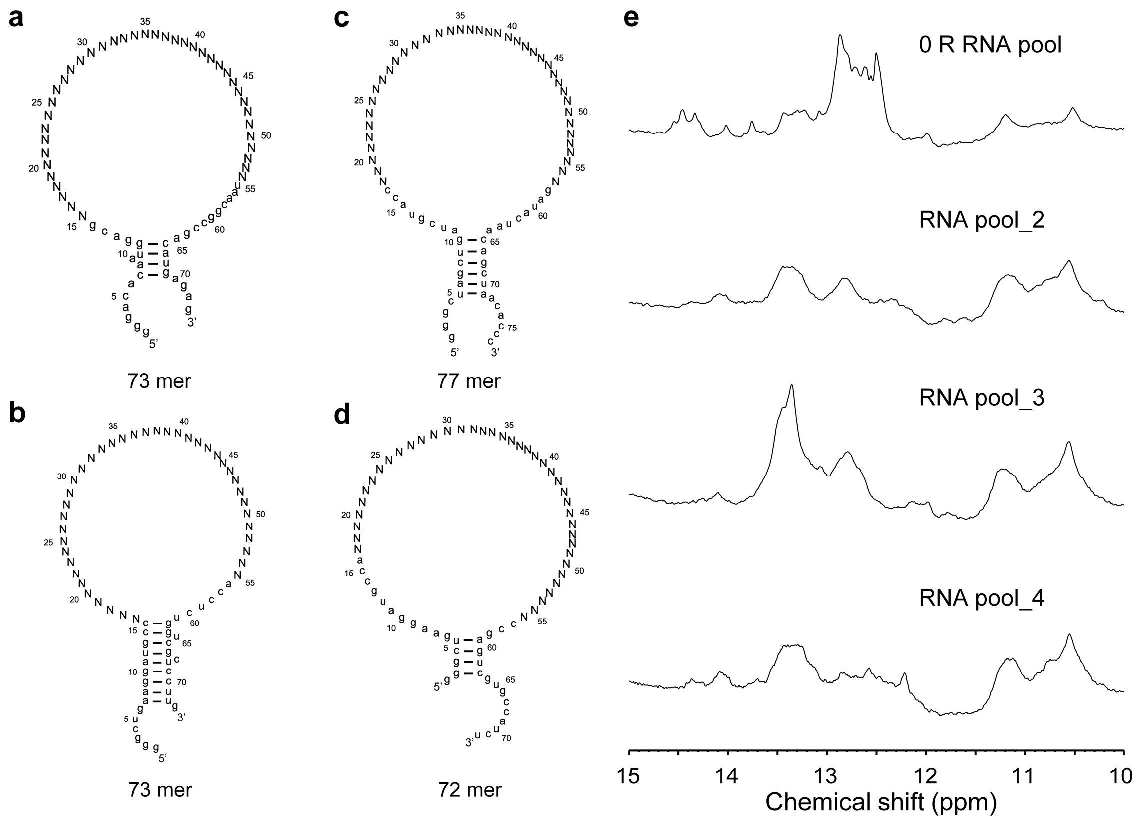

Supplementary Figure S7. Comparison of 1D imino proton spectra between various 0R RNA pools. The predicted secondary structure of the 0R RNA pool (**a**), RNA pool_2 (**b**), RNA pool_3 (**c**), and RNA pool_4 (**d**). Lower-case letters indicate nucleotides derived from the primer-binding sequences. 1D imino proton spectra of the 0R RNA pool (top), RNA pool_2 (second from top), RNA pool_3 (third from top), and RNA pool_4 (bottom) (**e**).

References

1. Dawson, W., Fujiwara, K., Futamura, Y., Yamamoto, K. & Kawai, G. A method for finding optimal RNA secondary structures using a new entropy model (vsfold). *Nucleosides Nucleotides Nucl. Acids* **25**, 171–189 (2006).

2. Dawson, W., Takai, T., Ito, I., Shimizu, K. & Kawai, G. A new entropy model for RNA: part III, Is the folding free energy landscape of RNA funnel shaped? *Journal of Nucleic Acids Investigation* **5**, 2652 (2014).

3. Amano, R.et al. Kinetic and Thermodynamic Analyses of Interaction between a High-Affinity RNA Aptamer and its Target Protein. *Biochemistry* **55**, 6221-6229 (2016).
